# Outbreak of *Achromobacter xylosoxidans* in an Italian Cystic fibrosis center: genome variability, biofilm production, antibiotic resistance, and motility in isolated strains

**DOI:** 10.3389/fmicb.2014.00138

**Published:** 2014-04-03

**Authors:** Maria Trancassini, Valerio Iebba, Nicoletta Citerà, Vanessa Tuccio, Annarita Magni, Paola Varesi, Riccardo V. De Biase, Valentina Totino, Floriana Santangelo, Antonella Gagliardi, Serena Schippa

**Affiliations:** ^1^Microbiology Section, Department of Public Health Sciences, Sapienza UniversityRome, Italy; ^2^Pediatrics Department, Regional Cystic Fibrosis Center, Sapienza UniversityRome, Italy

**Keywords:** *Achromobacter xylosoxidans*, Cystic fibrosis, antimicrobial resistance, motility, RAPD, biofilm

## Abstract

Cystic fibrosis (CF) patients have chronic airway infection and frequent exposure to antibiotics, which often leads to the emergence of resistant organisms. *Achromobacter xylosoxidans* is a new emergent pathogen in CF spectrum. From 2005 to 2010 we had an outbreak in *A. xylosoxidans* prevalence in our CF center, thus, the present study was aimed at deeply investigating virulence traits of *A. xylosoxidans* strains isolated from infected CF patients. To this purpose, we assessed *A. xylosoxidans* genome variability by randomly amplified polymorphic DNA (RAPD), biofilm production, antibiotic resistances, and motility. All *A. xylosoxidans* strains resulted to be biofilm producers, and were resistant to antibiotics usually employed in CF treatment. Hodge Test showed the ability to produce carbapenemase in some strains. Strains who were resistant to β-lactamics antibiotics, showed the specific band related to metal β-lactamase (bla_IMP-1_), and some of them showed to possess the integron1. Around 81% of *A. xylosoxidans* strains were motile. Multivariate analysis showed that RAPD profiles were able to predict Forced Expiratory Volume (FEV1%) and biofilm classes. A significant prevalence of strong biofilm producers strains was found in CF patients with severely impaired lung functions (FEV1% class 1). The outbreak we had in our center (prevalence from 8.9 to 16%) could be explained by an enhanced adaptation of *A. xylosoxidans* in the nosocomial environment, despite of aggressive antibiotic regimens that CF patients usually undergo.

## Introduction

In sputa of patients with Cystic fibrosis (CF), the Gram-negative *Pseudomonas aeruginosa* and the Gram-positive *Staphylococcus aureus* are the most frequently found pathogens. Recently, new bacterial species have also emerged, such as *Burkholderia cepacia* complex, *Stenotrophomonas maltophilia*, and *Achromobacter xylosoxidans* (Dakin et al., 2002; Rajan and Saiman, 2002; Saiman and Siegel, 2004; Vonberg and Gastmeier, 2005; Spicuzza et al., 2009; Hansen et al., 2010). *A. xylosoxidans* is an environmental Gram-negative bacillus with harmful properties in immuno-compromised hosts. It is difficult to be correctly identified, and suffers from a confusing nomenclature (Liu et al., 2002; Hauser et al., 2011). *A. xylosoxidans* is increasingly being isolated from sputa of CF patients (Zemel et al., 2000; Liu et al., 2002): its current prevalence ranges from 2 to 11% (Tan et al., 2002; Raso et al., 2008; Magni et al., 2010; Lambiase et al., 2011). Recently, a Brazilian study reported a prevalence of 21.8% (Pereira et al., 2011). Infection is frequently transient, although approximately 2.3% of CF patients are chronically infected (Tan et al., 2002). Relatively little is known about the clinical significance of *A. xylosoxidans* in CF patients, but it was found that infection occurs in CF patients with advanced lung disease (Lambiase et al., 2011): thus, additional studies are necessary to determine the aetio-pathological role *A. xylosoxidans* in CF. To this end, we decided to study some characteristics of *A. xylosoxidans* in selected strains isolated from CF patients, such as: genomic variability, biofilm production, antibiotic resistance, and motility. The last three bacterial features are considered important prerequisites for *in vivo* colonization and infection, and recent evidence report on their interdependence (Molin and Tolker-Nielsen, 2003; Hoffman et al., 2005; Shrout et al., 2011; Boles and Horswill, 2012). During chronic lung infection of CF patients, bacteria can survive under the challenging selective pressure imposed by both immune system and antibiotic regimens, developing both antibiotic resistances and biofilm formation (Macfarlane et al., 2011; Sibley and Surette, 2011; Sibley et al., 2011; Bragonzi et al., 2012). Furthermore, many bacterial species become highly motile thanks to enhanced production of flagella and pili (Henrichsen, 1983; Jarrell and McBride, 2008), also within established biofilms (Houry et al., 2012), increasing the efficiency in nutrient acquisition, the ability in avoiding toxic compounds, and the colonization of new available patches (Amiel et al., 2010; Sibley and Surette, 2011). Due to the doubled prevalence of CF patients colonized by *A. xylosoxidans* in our CF center, the present study was aimed at characterizing 57 strains of *A. xylosoxidans* isolated from CF patients and non-CF patients by: (i) genomic variability assessed by RAPD; (ii) ability to produce biofilm on abiotic surface; (iii) antibiotic resistance patterns; (iv) motility assay. Multivariate analysis was employed to cross-correlate all collected data.

## Materials and methods

### Patients

From January 2008 to January 2010, our laboratory isolated 225 strains of *A. xylosoxidans* from respiratory samples (i.e., sputum and tracheal aspirated) of 80 CF patients attending the CF Centre of the Pediatric Department of Policlinico Umberto I of Rome and from 5 non-CF patients. For the present study, we selected 52 strains from 34 CF patients, taken within the aforementioned group of 80, for whom *A. xylosoxidans* strains were isolated more than once. In addition, five strains were isolated from 4 non-CF patients: three strains from respiratory samples of 2 patients with a genetic respiratory disorder (Kartagener syndrome), and other two strains from 2 blood samples. Thus, the total number of selected *A. xylosoxidans* strains for this study was 57 from overall 38 patients. We divided the 34 CF selected patients into the following three Forced Expiratory Volume (FEV1%) classes (following the European Respiratory Society's criteria): class 3, mild obstruction or normal (≥70%); class 2, moderate obstruction (>40 and <70%); and class 1, severe obstruction (≤40%). A further subdivision was based on the chronic or intermittent presence of *A. xylosoxidans*. Chronicity was considered when there was a sputum culture positive for *A. xylosoxidans* in at least three occasions over a 6-month period, as previously suggested (Tan et al., 2002). In Table 1 are summarized patients' demographics and clinical features. All patients were involved in the study after providing written consent. The study protocol was approved by the Committee on Ethical Practice of the Policlinico Umberto I, Rome, Italy.

**Table 1:** **Patients' demographics and strains characteristics**.

**Patient**	**Age (year)**	**FEV1% class**	**Weight (kg)**	**Height (m)**	**BMI**	**Strain^c^**	**Biofilm class**	**Chronicity**	**ResistANces^e^**	**Swimming (mm)**
1	20	2	48.0	1.70	16.6	129	M	1	ATM, GM, NN	17.7
		1	47.0	1.70	16.2	157	W	1	ATM, GM, NN	23.7
2 (K)^a^	42	na^b^	76.5	1.82	23.1	232	S	1	AN, ATM, FEP, CIP, GM, MER, PIP, TIM, NN	13.0
3	56	2	80.0	1.65	29.4	66	W	0	AN, ATM, FEP, CIP, GM	26.3
4	6	na	19.2	1.10	15.9	180	M	1	AN, ATM, FEP, CIP, GM, NN	24.3
5	44	1	60.0	1.74	19.8	128	S	1	AN, ATM, FEP, CIP, CS, GM, MN, NN	na
		2	60.0	1.74	19.8	147	W	1	AN, FEP, CIP, GM, NN	22.7
		3	60.0	1.74	19.8	248	S	1	AN, ATM, FEP, CIP, GM, NN	na
6	36	1	41.0	1.55	17.1	35	M^d^	0	AN, ATM, FEP, CIP, GM, NN	35.7
		2	50.0	1.55	20.8	241	S	0	ATM, FEP, CIP, GM, MER, MN, NN	28.3
7	34	1	44.0	1.65	16.2	145	S^d^	0	AN, ATM, FEP, CIP, CS, GM, MN, NN	na
8	32	3	62.8	1.69	21.9	53	W	1	AN, ATM, GM	52.3
		2	61.0	1.69	21.3	228	S	1	AN, ATM, FEP, CIP, GM, PIP, NN	na
		2	61.0	1.69	21.3	229	W	1	AN, ATM, FEP, CIP, GM, MN, NN	23.7
9	23	1	45.0	1.78	14.2	209g	S	1	ATM, MER	31.0
		1	45.0	1.78	14.2	209p	S	1	FEP, CIP, MER	28.0
10	15	3	40.8	1.51	17.9	144	S	1	AN, ATM, FEP, GM, PIP, NN	29.3
11	48	1	52.0	1.63	19.6	4	S	1	AN, ATM, FEP, CAZ, CIP, GM, IPM, MER, PIP, TIM, NN	23.7
		na	55.0	1.64	20.5	276	S	1	AN, ATM, FEP, CIP, GM, NN	56.3
		na	55.0	1.64	20.4	277	S	1	AN, ATM, FEP, CIP, GM, NN	52.3
12	35	1	68.4	1.72	23.1	77	M	0	AN, ATM, FEP, CIP, GM, NN	39.7
		1	68.4	1.72	23.1	287	M	0	AN, ATM, FEP, CIP, CS, GM, MN, NN	50.3
13	5	na	21.7	1.10	17.8	162	W	0	AN, ATM, CS, GM	52.3
14	19	2	58.3	1.70	20.2	84	S	1	ATM, GM	na
15	7	3	19.6	1.20	13.6	101p	M	0	AN, ATM, CS, GM, NN	9.7
16	23	1	40.0	1.56	16.4	266	S	1	GM, NN	14.7
		1	40.0	1.56	16.4	278	S	1	AN, GM, NN	14.0
17	24	1	52.0	1.61	20.1	252	S	0	AN, ATM, CIP, GM, MN, NN	na
18	21	2	60.0	1.67	21.5	222	W	0	ATM, CIP	56.3
19	34	1	na	na	na	116	S	1	AN, ATM, CIP, GM, NN	23.0
		na	48.5	1.70	16.8	267	S	1	AN, ATM, FEP, CIP, CS, GM, NN	21.3
20	8	2	21.3	1.21	14.5	207	M	0	GM	30.7
21	37	2	58.0	1.67	20.8	251	S	1	AN, ATM, FEP, CAZ, CIP, GM, IPM, TIM, NN	61.7
22	6	3	23.0	1.23	15.2	153	S	0	–	na
23 (B)^a^	61	na	na	na	na	291	S	0	ATM, GM	31.0
24 (B)^a^	51	na	na	na	na	230	S	0	AN, ATM, CS, GM	43.7
25	22	2	68.0	1.88	19.2	167p	S	0	AN, ATM, FEP, CIP, GM, MN, PIP, NN	na
26	21	3	53.0	1.68	18.8	226	M	1	AN, ATM, FEP, CIP, GM, MN, NN	42.7
27	24	3	40.0	1.51	17.5	133	S	0	AN, ATM, FEP, CIP, CS, GM, NN	na
		2	40.0	1.51	17.5	231	M	0	AN, ATM, FEP, CS, GM	9.3
28	26	1	48.0	1.56	19.7	68	S	0	AN, ATM, GM, NN	35.0
29	22	3	67.0	1.82	20.2	247	S	0	ATM	40.3
30	21	3	62.6	1.73	20.9	225	S	0	AN, ATM, FEP, CIP, GM, NN	55.7
31	44	1	49.0	1.56	20.1	24	S	0	ATM, GM, MN, NN	20.3
32	14	3	56.0	1.60	21.8	49	M	1	AN, ATM, GM	54.0
		3	56.5	1.60	22.1	50	M	1	ATM	53.0
33	43	2	52.0	1.67	18.6	275	M	1	AN, ATM, FEP, CIP, GM, NN	8.3
34	1	na	8.6	0.72	16.3	215	M	0	ATM, NN	21.7
35	27	2	55.0	1.61	21.2	90	M	0	AN, ATM, FEP, CIP, CS, GM, NN	28.3
		1	61.6	1.60	24.1	237	S	0	AN, ATM, FEP, CIP, GM, NN	50.3
36 (K)	34	na	59.0	1.63	22.2	175	S	0	AN,. ATM, FEP, CIP, GM, NN	33.7
37	36	1	49.6	1.61	19.1	213	S	1	AN, FEP, CIP, GM, MN	11.7
38 (K)	26	na	na	na	na	67	S	0	AN, ATM, GM	43.0
39	30	3	52.7	1.65	19.3	249	M	1	AN, FEP, CS, IPM, MER, PIP	22.7
		3	52.7	1.65	19.3	250	S	1	AN, ATM, FEP, CIP, GM, IPM, TIM, NN	na
		3	54.9	1.65	20.2	218g	M	1	AN, ATM, FEP, CIP, GM, NN	55.0
		3	54.9	1.65	20.2	218p	S	1	FEP, CIP, IPM, MN	na

a *K, Kartagener syndrome; B, blood culture*.

b *na, not available*.

c *Letters g and p stand for different morphologies of the A. xylosoxidans colony grown on BCSA*.

d *Biofilm class was assessed after PLS-DA model*.

e *Resistances were determined by automated Vitek 2 system: ATM, aztreonam; GM, gentamicin; AN, amikacin; NN, tobramycin; FEP, cefepime; CIP, ciprofloxacin; CS, colistin; MN, minociclin; MER, meropenem; PIP, piperacillin; IPM, imipenem; TIM, ticarcillin-clavulanic acid; CAZ, ceftazidime; and TZP, piperacillin + tazobactam*.

### Microbial identification

API 20NE system (bioMérieux, Marcy l'Etoile, France) and automated Vitek2 system (bioMérieux, Marcy l'Etoile, France) were used for strains identification. Oxidase activity was checked with dimethyl-paraphenylenediamine disks (bioMérieux, Marcy l'Etoile, France). Results obtained from API 20NE tests and oxidase reactions were further interpreted with the Apilab Plus software package (bioMérieux, Marcy l'Etoile, France). In order to avoid misidentification of Gram negative bacilli, isolated from CF patients, we performed species-specific PCR for all *A. xylosoxidans* strains. Collected strains were cryopreserved at −80°C before use. Antibiotic resistances assays for all *A. xylosoxidans* strains were provided by automated Vitek2 system.

### DNA extraction

*A. xylosoxidans* DNA extraction was performed by use of a Wizard genomic DNA purification kit (Promega Corporation, Madison, WI) following manufacturer's instructions. DNA was finally quantified by spectrophotometer at 260 nm, and its quality assayed by 260/280 nm ratio.

### Species-specific PCR assay

Species-specific PCR was performed as described elsewhere (Hogardt et al., 2007). Negative and positive control PCRs were employed for every experiment. PCR products were visualized by electrophoresis in a 2% agarose gel (Invitrogen Corporation, CA), stained with ethidium bromide (EtBr; Invitrogen Corporation), and captured with a DigiDoc-It (UVP, Cambridge, United Kingdom) photographic system. Bands of 163 bp were considered positive for *A. xylosoxidans* identification.

### RAPD typing

The RAPD amplification mixture and cycling conditions were described elsewhere (Lambiase et al., 2006). The primer used was the 270 (5′-TGCGCGCGGG-3′). RAPD products were separated by electrophoresis in 1.5% agarose gel (Invitrogen Corporation, CA). Molecular size markers (Invitrogen Corporation) and negative control were included in all gels. Gels were stained with EtBr (at 0.5 μM) (Invitrogen Corporation) and captured with a DigiDoc-It (UVP) photographic system.

### Biofilm production assay

Overnight cultures of *A. xylosoxidans* in Trypticase Soy Broth (Becton Dickinson) at 37°C in dynamic conditions (90 rpm), were diluted into fresh TSB to reach OD_550_ = 1 (corresponding to 1 × 10^9^ CFU/ml). After diluting 1:100 the bacterial culture, 200 μl were used to inoculate sterile flat-bottom polystyrene tissue culture 96-wells plates, followed by a 48 h incubation at 37°C. Non-adherent bacteria were removed by washing three times with sterile Phosphate Buffered Saline (PBS). Wells were stained at room temperature for 5 min with 200 μl of 1% Crystal Violet solution, then rinsed with distilled water and dried at 37°C for 30 min. Stained biofilm were dissolved adding 250 μl of 33% glacial acetic acid for 15 min. The optical density (OD) of each well was measured at 570 nm using a microtiter-plate reader (Multiskan EX, Thermo Scientific, Massachusetts, USA). *A. xylosoxidans* strains were divided following Stepanovic method (Stepanovic et al., 2007), into different biofilm producers classes, named: N, no biofilm producer; W, weak biofilm producer; M, moderate biofilm producer; and S, strong biofilm producer.

### Motility assay

Swimming assay was performed in “Swim plates” [tryptone broth (10 g/l tryptone (Difco)-5 g/l NaCl) containing 0.3% (wt/vol) agarose (GIBCO/BRL)]. LB agar Overnight culture of isolated strains identified as *A. xylosoxidans*, were used to inoculate Swim plates, and incubated at 30°C for 12–14 h (Rashid et al., 2000). Diameter of resulting concentric ring, expressed in millimetres, was used to define the motility of a specific *A. xylosoxidans* strain.

### PCR detections of resistance determinants

The presence of Class 1 Integron was tested by PCR, by means of specific primers: 5′-CS (GGC ATC CAA GCA GCA AGC) and 3′-CS (AAA GCA GAC TTG ACC TGA) (Levesque et al., 1995; Neuwirth et al., 2006). Carbapenemase determinants *bla*IMP, *bla*VIM, and β-lactamase determinants *bla*VEB plus *bla*OXA-1 were also assayed by PCR. Primer used were: IMP-1 F (5′CAT GGT TTG GTG GTT CTT GT 3′) and IMP-_1_ R (5′ATA ATT TGG CGG ACT TTG GC 3′) for *bla*IMP (Yum et al., 2002); VIM-F (5′-AGT GGT GAG TATCCG ACA G-3′) and VIM-R (5′-ATG AAA GTG CGTGGA GAC-3′) for *bla*VIM_1_ (Tsakris et al., 2000); VIM-2A (5′-ATGTTCAAACTTTTGAGTAGTAAG-3′) and VIM-2B (5′CTACTCAACGACTGAGCG-3′) for *bla*VIM-_2_-like genes (Poirel et al., 2000); VEB-1 F (5′ CCA GAT AGG AGT ACA GAC 3′) and VEB-1 R (5′ GAC TCT GCA ACA AAT ACG C 3′) for *bla*VEB_1_ (Neuwirth et al., 2006); OXA-1 F (5′CTT GAT TGA AGG GTT GGG CG-3′) and OXA-1 R (5′AGC CGT TAA AAT TAA GCC C-3′) for *bla*OXA-_1_ (Shin et al., 2005).

### Data analysis

#### Agglomerative hierarchical classification (AHC)

AHC, an unsupervised method, was performed on RAPD profiles by means of a binary matrix generated by the presence/absence of RAPD bands, using Doc-It LS software (UVP), and the subsequent dendrogram was generated with XLStat 7.5 (Addinsoft), using a Euclidean distance dissimilarity matrix and the agglomeration method of Ward.

#### Factorial discriminant analysis (FDA)

FDA, a supervised method closely linked to multivariate analysis of variance, was employed by means of XLStat 7.5 software (Addinsoft). Explanatory variables were automatically verified to be linearly independent by calculating the multiple correlation of each variable with all the others. Fisher's test was used to compare patients' clinical features or characteristics of selected *A. xylosoxidans* strains with RAPD profiles: a *P* value less than or equal to 0.05 was considered statistically significant.

## Results

### *Achromobacter xylosoxidans* isolates

Overall 39 patients referring to the Regional CF Centre, “Policlinico Umberto I” hospital (Rome), were enrolled. Thirty-four above the 39 patients were affected by CF, while the remaining non-CF subjects had a different disease (three patients with Kartagener syndrome, that involves an impairment in epithelial cilia movement, and other two patients from an oncologic unit). Patients with CF and Kartagener syndrome gave sputa samples, while subjects from oncologic unit gave blood samples. All CF patients were not permanently convalescing at the Policlinico Umberto I hospital, but underwent a visit each 6 months, or when specifically required by the physician. Patients' antibiotic treatments were as follows (taking into consideration the closer to *A. xylosoxidans* strains isolation from each CF patient): colimycin 41.18% (14/34), sulfametoxazole + trimethoprim 17.65% (6/34), tobramycin 11.76% (4/34), amoxicillin + clavulanic acid 8.82% (3/34), levofloxacin 8.82% (3/34), amikacin 5.88% (2/52), ceftriaxone 2.94% (1/34), linezolid 2.94% (1/34). All CF patients went through aforementioned antibiotic treatments for a period of 6 months before *A. xylosoxidans* sampling under these conditions: colimycin aerosolized 2 flasks/day every 2 days, sulfametoxazole + trimethoprim orally provided every 2 days, tobramycin aerosolized 300 mg/day, amoxicillin + clavulanic acid orally provided every 2 days, levofloxacin provided intravenously 300 mg/day, amikacin aerosolized 1 flask/day, ceftriaxone provided intravenously 1 dose/day, linezolid orally provided every 2 days. Patients' demographics, along with number and characteristics of *A. xylosoxidans* isolates, were reported in Table 1. The isolation range was 1–4 bacterial strains per patient, with a total of 57 *A. xylosoxidans* strains. At the same time of isolation, data about the Forced Expiratory Value (FEV), expressed as FEV1%, and the Body Mass Index (BMI), were collected. In the present study, spanning from January 2008 to January 2010, we had 80 patients *A. xylosoxidans*-positive patients above a total of 500, with a prevalence of 16%: 34/80 were chronically infected, and we were able to isolate 225 strains. In respect to our previous study (Magni et al., 2010), we had a significantly higher prevalence of *A. xylosoxidans*-positive patients: from 8.9 to 16% (χ^2^ = 10.18, *P* = 0.0014).

### RAPD-PCR

**Figure 1:**
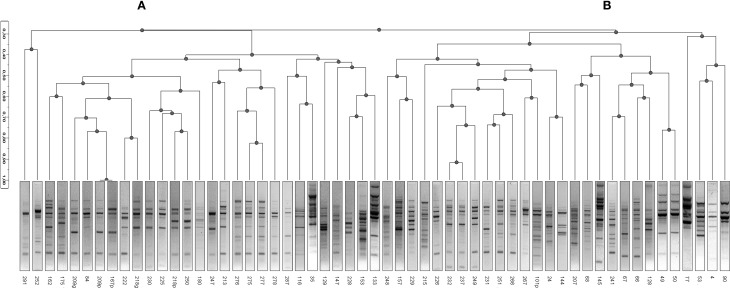
**Ascendent Hierarchical Clusterization (AHC)**. A dendrogram based on RAPD profiles of *A. xylosoxidans* strains was generated by means of inverse Euclidean distance dissimilarity matrix and agglomeration method of Ward. Two clusters are visible (**A** and **B**) based upon a threshold set at 28% of similarity. No known variable was responsible in defining such a cluster formation (for all variables, *P* > 0.05).

The 57 strains of *A. xylosoxidans* were characterized for their genomic asset by the Randomly Amplified Polymorphic DNA (RAPD) technique, useful to look for genetic relationships among the isolated strains and for discerning strains within a species. In Figure 1 was reported the dendrogram obtained by Ascendent Hierarchical Clusterization (AHC), a non-supervised statistical method to ascertain genomic relatedness among RAPD profiles obtained. The dendrogram showed two major clusters (A and B): the first cluster (A) grouped 29/57 *A. xylosoxidans* isolates, while the second one (B) grouped 28/57 bacterial strains (Figure 1). Intra-cluster mean similarity was calculated by Dice index, as described in Materials and Methods, and gave a result of 45.9 ± 0.8% for cluster A, while it was 41.7 ± 0.8% for cluster B, and this difference was significant (Mann-Whitney, *P* = 0.00078).

### Biofilm production

In our assay conditions, all strains were able to produce biofilm on abiotic surface (96-well plate) (Table 1). On the basis of biofilm amount produced, assessed at OD_570_ accordingly to Stepanovic (Stepanovic et al., 2007), *A. xylosoxidans* strains were divided into three different groups: strong (S), moderate (M), and weak (W). As reported in Table 1, 33/57 (57.9%) strains were strong biofilm producers, 15/57 (26.3%) strains felt into moderate class, while 7/57 (12.3%) strains were weak producers. Two strains above 57 (3.5%), namely 35 and 145, showed OD_590_ values intermediate between strong and moderate produces. Those strains were consequently placed in the class moderate and strong, respectively, on the basis of their RAPD profiles (see results of statistical correlations).

### Motility assay

Forty-six above 57 *A. xylosoxidans* strains (80.7%) showed a swimming phenotype (Table 1), with the formation of a concentric ring with different diameters, expressed in millimetres. Eleven *A. xylosoxidans* isolates resulted negative for swimming motility. No swarming as well as no twitching phenotype were found among the isolated strains of *A. xylosoxidans* (data not shown).

### Antibiotic resistance phenotype

**Figure 2:**
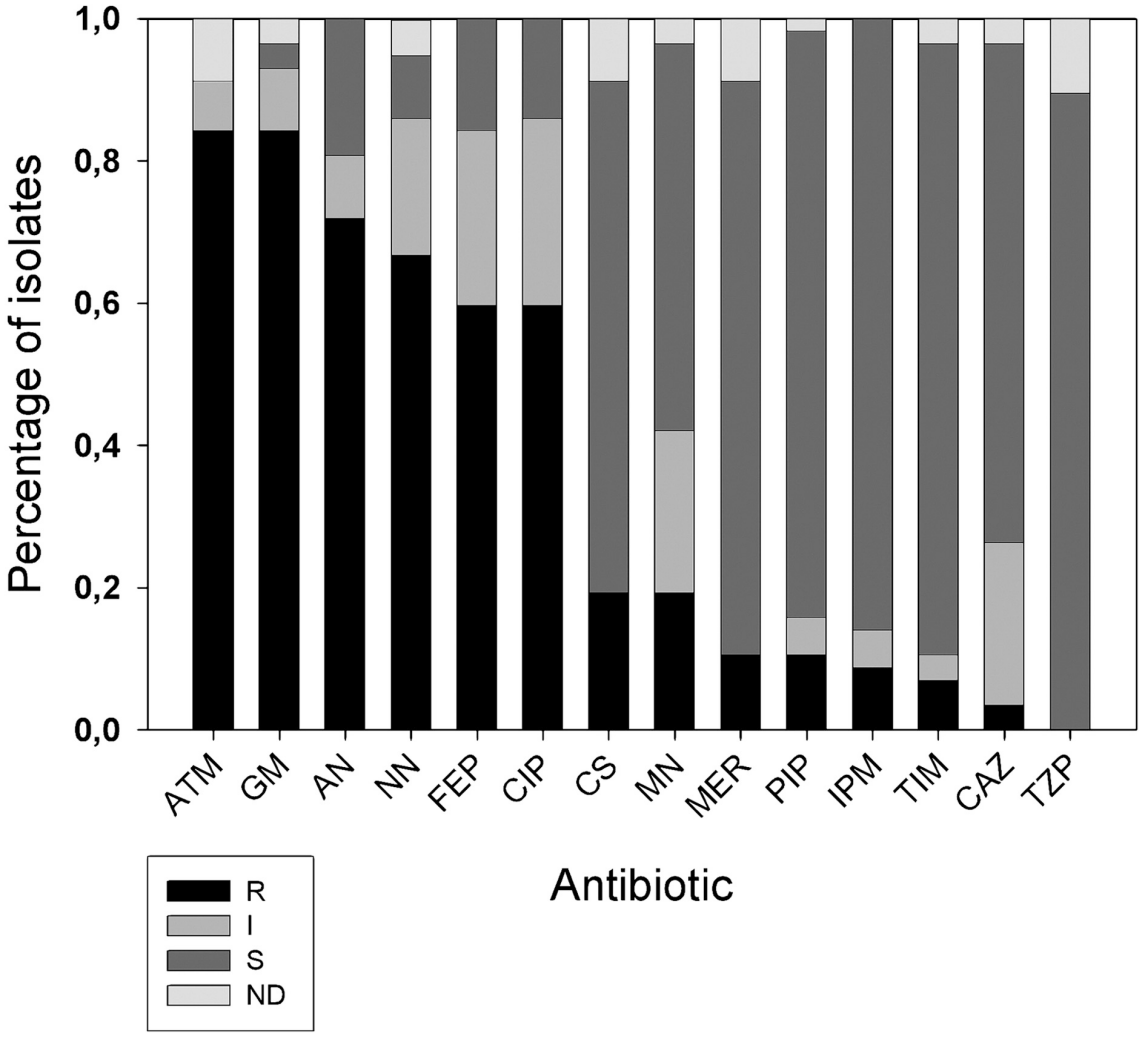
**Distribution of antibiotic resistances among *A. xylosoxidans* isolates**. Fourteen antibiotics (*x* axis) were tested with Vitek 2 instrument: ATM, aztreonam; GM, gentamicin; AN, amikacin; NN, tobramycin; FEP, cefepime; CIP, ciprofloxacin; CS, colistin; MN, minociclin; MER, meropenem; PIP, piperacillin; IPM, imipenem; TIM, ticarcillin clavulanate; CAZ, ceftazidime; and TZP, piperacillin+tazobactam. On *y* axis is the fraction of isolates normalized to one. R, resistant; I, intermediate; S, susceptible; ND, indeterminate.

All 57 *A. xylosoxidans* isolates were tested for 14 antibiotic resistances by means of Vitek2 instrument. Results showed (Figure 2) high frequency of resistances to aztreonam (84%), gentamicin (84%), amikacin (75%), tobramycin (73%), cefepime (60%) e ciprofloxacin (60%). Hodge Test showed that 2/11 of the carbapenem resistant strains were able to produce carbapenemase. 3/57 strains resistant to β-lactam antibiotics showed to possess the specific band of the integron1, and 7 the specific band related to metal β-lactamase (bla_IMP−1_). Of these last 7 strains, three showed to possess the specific band of the integron1 meropenem (MER), piperacillin (PIP), imipenem (IPM), ticarcillin clavulanate (TIM), ceftazidime (CAZ). Taking into consideration the antibiotic exposure of the 52 selected *A. xylosoxidans* strains among CF patients, we found these percentages of prevalence: Amoxicillin + clavulanic acid 9.62% (5/52), levofloxacin 9.62% (5/52), ceftriaxone 3.85% (2/52), tobramycin 11.54% (6/52), colimycin 38.46% (20/52), linezolid 3.85% (2/52), sulfametoxazole + trimethoprim 19.23% (10/52), amikacin 3.85% (2/52).

### Data correlations

#### RAPD—FEV1%

**Figure 3:**
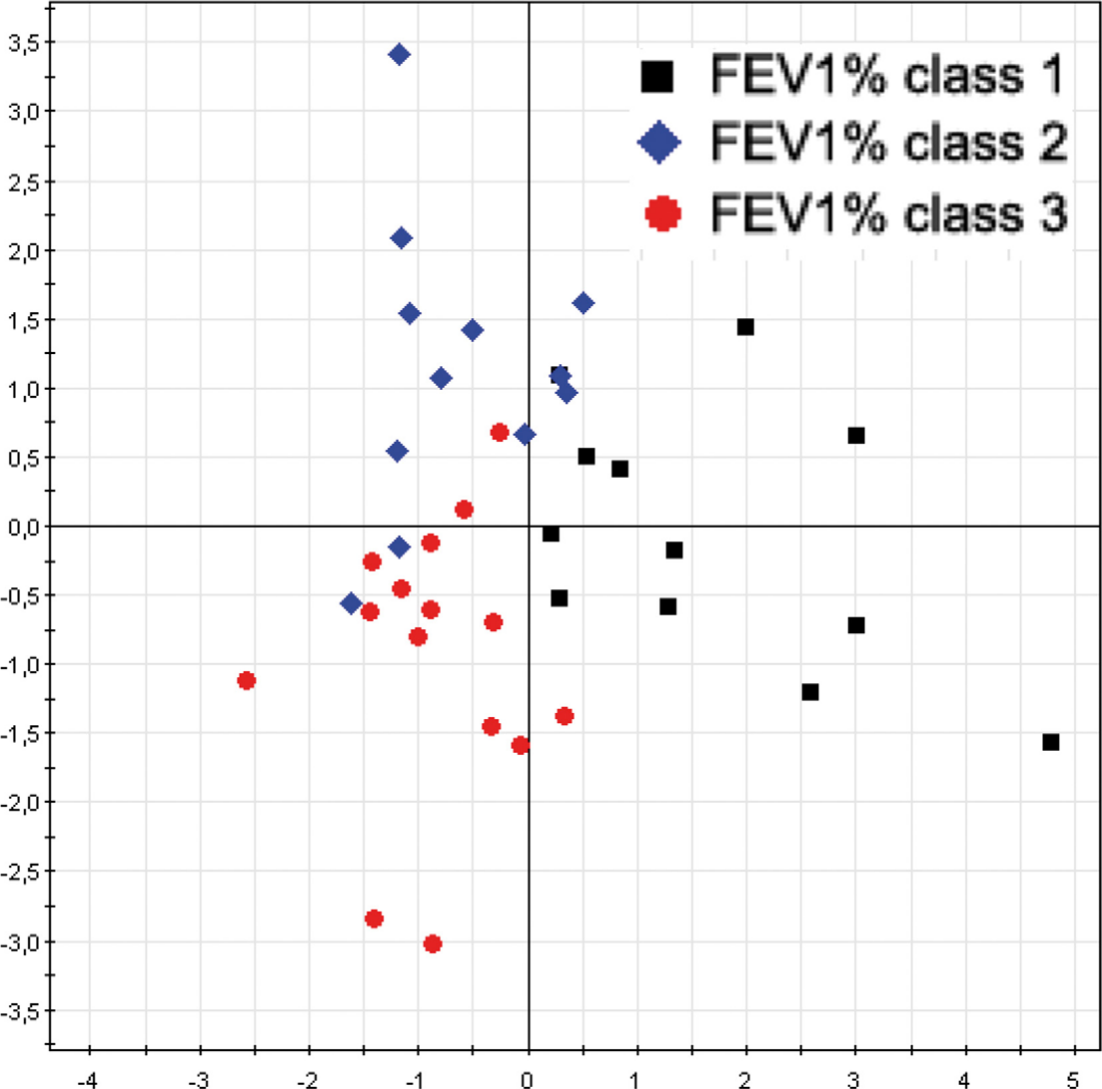
***A. xylosoxidans* RAPD profiles grouped by FEV1% class**. The overall data variation was described by the factorial axes t[1] and t[2]. The predictability of the model, in dividing FEV1% classes, was 84.62% (Fisher's *P* = 5.2^*^10^−10^).

In order to confirm the results already obtained in a previous our study (Magni et al., 2010) we performed a correlation analysis between RAPD profiles of the new 57 *A. xylosoxidans* strains and patients' FEV1% classes. A multivariate statistical approach, performed with Factorial Discriminant Analysis (FDA) algorithm, showed a separation between the FEV1% classes (Figure 3), with a model predictability of 84.62% (Fisher's *P* = 5.2^*^10^−10^). The analysis correctly classified 9/12 (75.0%) of *A. xylosoxidans* strains isolated from CF patients with predicted FEV1% class 1, 14/15 (93.3%) of the strains isolated from CF patients with predicted FEV1% classes 3, and 10/12 (83.3%) of the strains isolated from CF patients with predicted FEV1% classes 2. Eighteen *A. xylosoxidans* strains with no predicted FEV1% class were classified among the three FEV1% classes on the basis of their RAPD profiles: 4 to class 1, 7 to class 2, and 7 to class 3. The FEV1% class assignment of these last 18 strains were in agreement with the clinical status of corresponding CF patients, as assessed by pediatric physicians.

#### RAPD—biofilm

**Figure 4:**
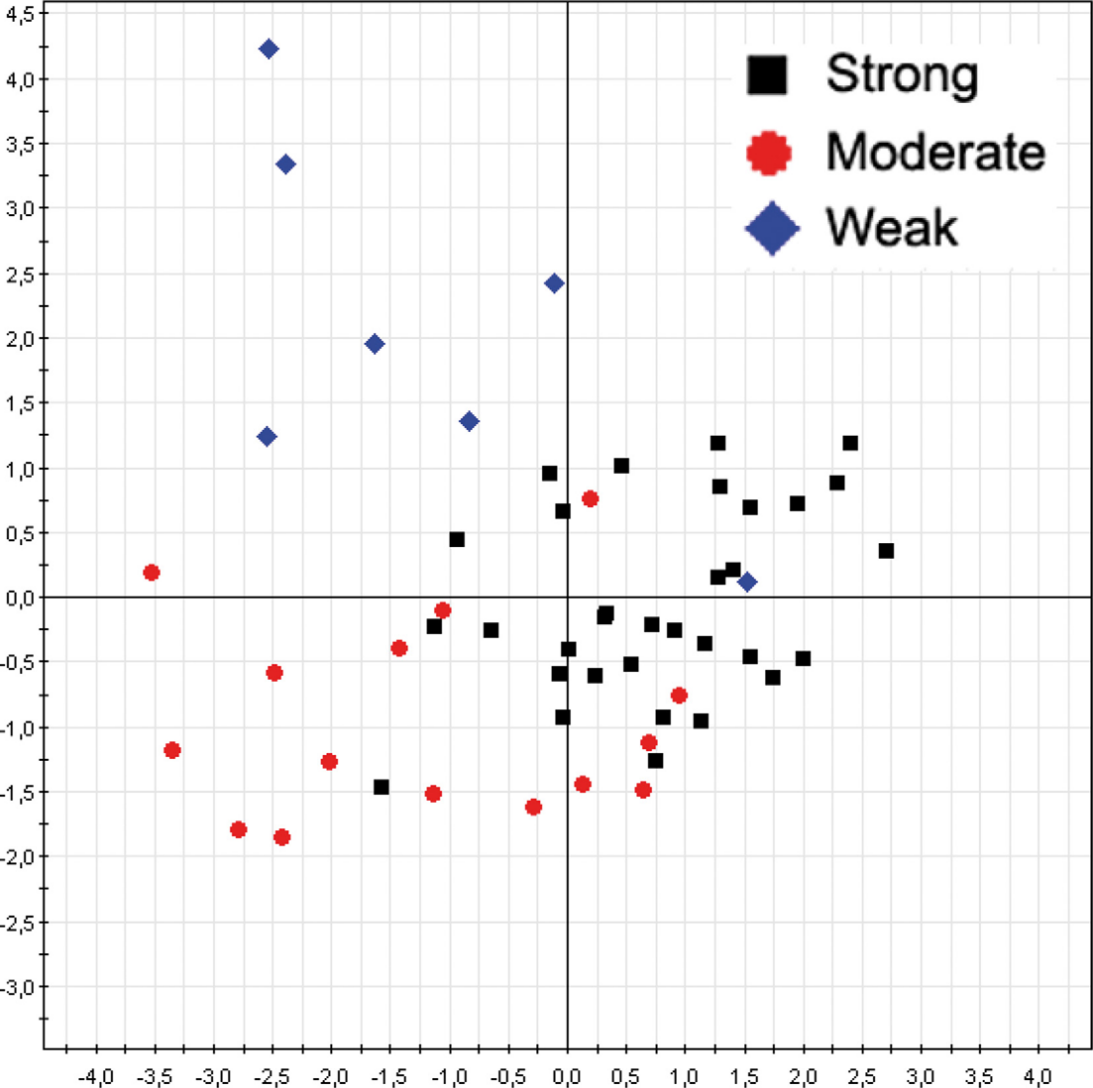
***A. xylosoxidans* RAPD profiles grouped by biofilm classes**. The overall data variation was described by the factorial axes t[1] and t[2]. The predictability of the model, in dividing biofilm classes, was 85.45% (Fisher's *P* = 5.4^*^10^−11^).

The same multivariate statistical approach of PLS-DA was employed to correlate *A. xylosoxidans* strains RAPD profiles and corresponding biofilm classes. The model showed a separation between the biofilm classes (Figure 4), with a model predictability of 85.45% (Fisher's *P* = 5.4^*^10^−11^). The analysis correctly classified 31/33 (93.9%) of strong biofilm producers, 10/15 (66.7%) of medium one, and 6/7 (85.7%) of weak biofilm producers. Two *A. xylosoxidans* strains without biofilm classification were classified upon their RAPD profiles in this way: one to strong class, and one to weak class. Furthermore, we studied the distribution of *A. xylosoxidans* strains, in relation to their biofilm production ability, between FEV1% classes. A significant prevalence of strong biofilm producers strains was found in FEV1% class 1 CF patients (FEV1% class 1 vs. FEV1% class 2, *P* = 0.0007, χ^2^ = 11.44; FEV1% class 1 vs. FEV1% class 3, *P* = 0.0046, χ^2^ = 8.02) (Table 2). No difference was found in the mean number of resistance traits among strains colonizing CF patients with different FEV1% value (data not shown). Similarly, no differences were found comparing mean number of resistance traits and biofilm producing ability among strains colonizing CF patients in a chronic or intermittent way (data not shown).

**Table 2:** **Distribution of collected strains among biofilm and FEV1% classes**.

	**Biofilm class**		
**FEV1% class**	***S* (***n*** = **33**)**	***M* (***m*** = **17**)**	***W* (***n*** = **7**)**
1	14	5	1
2	6	4	4
3	8	4	1
0	5	4	1

## Discussion

In a previous paper (Magni et al., 2010) we showed an *A. xylosoxidans* prevalence of 8.9% in our Regional CF center, referred to a time window from January 2005 to January 2007. In that paper we reported that above overall 450 patients, 40 were affected by *A. xylosoxidans*, 16 were chronically infected, and we isolated 106 strains. In the present study, spanning from January 2008 to January 2010, we found an almost doubled prevalence of *A. xylosoxidans*-positive patients (16%), meaning a possible outbreak in our center. Due to the doubled prevalence found, we focused our attention on well-known virulence traits able to enhance bacterial colonization. It was proposed how biofilm formation, antibiotic resistance acquisition, and motility could be interdependent (Molin and Tolker-Nielsen, 2003; Hoffman et al., 2005), and recent evidence support such hypothesis (Shrout et al., 2011; Boles and Horswill, 2012). We studied the abovementioned virulence factors in 57 CF and non-CF clinical isolates of *A. xylosoxidans*, in order to investigate their role in this emerging pathogen: this is the first report on biofilm formation and motility of *A. xylosoxidans*. Genomic fingerprint assessed by RAPD analysis showed a significant division into two major clusters (Figure 1). Within such clusters all features considered (BMI, FEV1%, chronicity, biofilm production, resistance determinants, motility) were randomly distributed: probably, other features not taken into account in this study are accountable for cluster formation. We showed a significant association between RAPD profiles, FEV1% and biofilm classes, meaning that different CF lung habitats would be able to select particular *A. xylosoxidans* genomic variants and biofilm producers. As reported in Table 2, we found a significant presence of strong biofilm producers within FEV1% class 1, underlining the influence of biofilm on clinical exacerbations in CF. Biofilm development is a common trait in CF patients at the surface of airway mucosae (Sibley and Surette, 2011; Sibley et al., 2011; Bragonzi et al., 2012): bacteria inside biofilms are well protected, developing antibiotic resistances even by means of horizontal genetic transfer (HGT) (Molin and Tolker-Nielsen, 2003). Interestingly, all our *A. xylosoxidans* strains having specific resistance integrons were strong biofilm producers, supporting the involvement of HGT in biofilm development (Ghigo, 2001; Traglia et al., 2012). In this view, the unique environmental *A. xylosoxidans* genome sequenced thus far (Strnad et al., 2011) showed the presence of two large plasmids with resistance genes. As found in *P. aeruginosa*, mechanisms other than HGT should be employed in biofilm formation, such as sub-inhibitory concentrations of aminoglycosides involving alterations of c-di-GMP levels (Hoffman et al., 2005). Interestingly, more than 70% of *A. xylosoxidans* strains were resistant to the three aminoglycosides (gentamicin, amikacin, tobramycin) tested by Vitek 2 instrument (Figure 2), paving the way of a double strategy (HGT and c-di-GMP) enrolled by *A. xylosoxidans* in producing biofilms within the lung environment to circumvent antibiotic pressure. We had a high prevalence of patients that underwent colimycin/colistin usage (41%) before *A. xylosoxidans* strain isolation, and we observed only a 20% of resistance to colistin assessed by Vitek2 (Figure 3): this discrepancy should be due to the short time of exposure (3 months) of the pathogen to the antibiotic. One could argue that a prolonged exposure should enhance the prevalence of this resistance determinant within the *A. xylosoxidans* population, but colimycin/colistin should be a good candidate to hinder an outbreak. Once established, bacteria are able to swim within the biofilm itself (Boles and Horswill, 2012; Houry et al., 2012), enhancing colonization of new patches and nutrient acquisition. Even if around 81% of our *A. xylosoxidans* strains showed swimming ability in “Swim plates,” no positive or negative correlation was found with biofilm production assessed by 96-wells plates (*R*^2^ = 5.7^*^10^−4^): thus, further studies will investigate on *A. xylosoxidans* motility within established biofilms by means of fluorescent dyes. This study evidenced how biofilm formation, antibiotic resistances acquisition, and motility showed by *A. xylosoxidans* species could enhance its survival in CF patients' lung.

### Conflict of interest statement

The authors declare that the research was conducted in the absence of any commercial or financial relationships that could be construed as a potential conflict of interest.
